# Low-Complexity Robust Adaptive Beamforming Based on INCM Reconstruction via Subspace Projection

**DOI:** 10.3390/s21237783

**Published:** 2021-11-23

**Authors:** Yanliang Duan, Xinhua Yu, Lirong Mei, Weiping Cao

**Affiliations:** 1 Guangxi Key Laboratory of Wireless Wideband Communication & Signal Processing, Guilin University of Electronic Technology at Guilin, Guilin 541004, China; duanyanliangdyl@163.com (Y.D.); yusilian@126.com (X.Y.); 2 The 54th Research Institute of China Electronics Technology Group Corporation, Shijiazhuang 050081, China; xymeilirong@163.com; 3 Science and Technology on Information Transmission and Dissemination in Communication Networks Laboratory, Shijiazhuang 050081, China

**Keywords:** robust adaptive beamforming, orthogonality, blocking matrix, interference-plus-noise covariance matrix reconstruction

## Abstract

Adaptive beamforming is sensitive to steering vector (SV) and covariance matrix mismatches, especially when the signal of interest (SOI) component exists in the training sequence. In this paper, we present a low-complexity robust adaptive beamforming (RAB) method based on an interference–noise covariance matrix (INCM) reconstruction and SOI SV estimation. First, the proposed method employs the minimum mean square error criterion to construct the blocking matrix. Then, the projection matrix is obtained by projecting the blocking matrix onto the signal subspace of the sample covariance matrix (SCM). The INCM is reconstructed by replacing part of the eigenvector columns of the SCM with the corresponding eigenvectors of the projection matrix. On the other hand, the SOI SV is estimated via the iterative mismatch approximation method. The proposed method only needs to know the priori-knowledge of the array geometry and angular region where the SOI is located. The simulation results showed that the proposed method can deal with multiple types of mismatches, while taking into account both low complexity and high robustness.

## 1. Introduction

Adaptive beamforming adjusts the weight vector according to the application environment to enhance the SOI by suppressing interference and noise, and it has been widely used in radar, sonar, microphone array speech processing, wireless communication, radio astronomy, and other areas [1,2,3]. Generally, the standard Capon beamformer (SCB) obtains the maximum-array-output-signal-to-interference-plus-noise ratio (SINR) if the covariance matrix and SOI SV are accurately known [4]. However, severe performance degradation may occur in the presence of SV and INCM mismatches due to the fact of array calibration errors, finite snapshots, and other factors, especially when the SOI component is presented in the INCM [5,6]. Therefore, various RAB algorithms have been proposed to ensure the robustness of beamformers over the past years. In general, these RAB methods can be classified into the following types [6,7]: diagonal loading (DL) technique, eigenspace-based (ESB) technique, uncertain-set based technique, and covariance matrix reconstruction-based technique.

DL is one of the most classical RAB methods for improving the robustness of a beamformer, which is derived by imposing a quadratic constraint either on the norm of the weight vector or on its SOI SV [8,9]. However, its major challenge is that it is difficult to choose the optimal DL level in different scenarios. To overcome this drawback, parameter-free methods in [4,10,11,12,13] can automatically compute the DL level without specifying any additional user parameters. Regrettably, these methods fail to provide satisfactory performance in high-input signal noise rates (SNRs).

The ESB technique is another type of traditional RAB method that is performed by projecting nominal SV onto the signal-plus-interference subspace to eliminate the arbitrary SV mismatch of SOI [14,15,16,17]. However, serious performance degradation will appear at low-input SNRs. In [15], a modified ESB method based on covariance matrix enhancement was proposed to improve the performance at low SNRs. In addition, the authors in [17] proposed a method to obtain the basis vector of signal subspace among the eigenvectors of the SCM. However, these methods perform poorly in the presence of large SV mismatch and high-input SNRs.

The uncertain-set-based technique utilizes a spherical or ellipsoidal uncertainty constraint setting on the nominal SV to estimate the SOI SV including the worst-case-based (WCB) method [5,18], doubly constrained method [10,19], probabilistically constrained method [20,21], and linear programming method [22]. However, these methods do not eliminate the SOI component from the SCM, and severe performance degradation will occur in the presence of high-input SNRs [7]. In addition, most of them need to solve the second-order cone programming (SOCP) problem, which leads to high complexity. Actually, the uncertain-set-based technique has been demonstrated to be equivalent to the DL method [6].

The above methods are mainly aimed at estimating SOI SV or SCM. Although these methods can improve the robustness of a beamformer, all still suffer from serious performance degradation at high-input SNRs. In order to overcome this drawback, a new type of RAB method based on INCM reconstruction has been developed in recent years. The authors of [23] firstly employ the SCB to estimate interference SV and reconstruct the INCM, but the power of interference was not accurately estimated. Gu, in [24], proposed an RAB method based on INCM reconstruction and SV estimation, where the INCM is reconstructed by integrating over the complement of the SOI angular region. However, the complexity is increased significantly. Subsequently, in [25,26], low-complexity shrinkage-based mismatch estimation (LOCSME) and the sparsity of the source distribution were used to significantly reduce the complexity. Unfortunately, these methods can achieve good performance only in certain conditions. To resist more types of mismatches, a new estimator for INCM based on interference SV and power estimation is presented in [6], and a QCQP problem with new inequality constraint was established to estimate the SOI SV. In [27], the authors constructed and solved a set of linear equations to obtain the estimation of interference power. Furthermore, the residual noise power was considered to improve the estimation accuracy of incident signal power in [28]. In [29], the iterative mismatch approximation method was employed to estimate the power and SV of all incident signals; then, these estimates were used to reconstruct the INCM. In [30], all nominal SVs were adjusted to an accurate version by a line search along the corresponding gradient vector. Together with the recorded power, the INCM was reconstructed. The Capon spectrum can be approximated as the power of noise when the SV mismatch is large enough. To overcome this drawback, the authors in [31] used the principle of maximum entropy power spectrum to reconstruct the interference and SOI covariance matrix by estimating all powers of incident signals. Different from the above INCM reconstruction-based methods, the authors in [32] reconstructed the INCM by projecting the interference subspace onto the received snapshots, which can effectively eliminate the SOI component and achieve good performance. In [33], the INCM reconstruction relies on using the average value of noise eigenvalues instead of the eigenvalue of the SOI to eliminate a noticeable part of the SOI. Ai et al. [34] presented an RAB algorithm for subspace projection and covariance reconstruction (SPCMR) that employs subspace projection and oblique projection to estimate the SOI SV and interference powers accurately. In [35], each SV was derived from the vector located at the intersection of two subspaces. Meanwhile, the estimate of each SV was given in a closed-form expression.

In this paper, a low-complexity RAB method based on INCM reconstruction and SOI SV estimation is proposed. Unlike previous methods, the INCM in the proposed method was reconstructed utilizing the orthogonality of subspace. First, based on the idea of the matrix filter in [36,37], the minimum mean square error criterion was employed to construct the blocking matrix. Then, we performed eigen-decomposition on the SCM and obtained the orthogonal projection matrix of the signal subspace. By projecting the blocking matrix onto the orthogonal projection matrix, a projection matrix was obtained. Subsequently, the INCM was reconstructed by replacing the eigenvector columns of the SCM, which can span to the signal subspace with the corresponding eigenvectors of the projection matrix. Finally, the SOI SV was estimated by employing the iterative mismatch approximation method presented in [29]. The theoretical analysis and simulation results demonstrated that the proposed method can efficiently deal with multiple types of mismatches.

The rest of this paper is organized as follows. The signal model and necessary background regarding the adaptive beamforming method is introduced in Section 2. In Section 3, the proposed RAB methods are described in detail, and the feasibility analysis of the blocking matrix is performed. The simulation results are provided in Section 4. Finally, conclusions are drawn in Section 5.

## 2. Signal Model and Background

Consider a uniform linear array (ULA) composed of M omnidirectional sensors that are illuminated by L+1 far-field uncorrelated narrowband signals, which consist of one SOI and L interferences. The array complex sample vector at time k can be presented as:

(1) $$x\left(k\right)=x_{s}\left(k\right)+x_{i}\left(k\right)+x_{n}\left(k\right),$$

where $x_{s}\left(k\right)=s_{0}\left(k\right)a_{0}$ and $x_{i}\left(k\right)=\overset{L}{\underset{l=1}{\sum }}s_{l}\left(k\right)a_{l}$, respectively, represent the M×1 vector of the SOI and interference signal component in the received data. $s_{l}\left(k\right)$ and $a_{l}\left(l=0,\ldots ,L\right)$ are the lth incident signal waveform and corresponding SV. $x_{n}\left(k\right)$ is additive complex Gaussian noise with a zero mean and a variance of $\sigma_{n}^{2}$, which is uncorrelated with all the other signals. In this paper, the sensors were spaced at half of the wavelength. The nominal SV from θ can be written as:

(2) $$\begin{array}{ll} a\left(\theta \right) & ={\left[1,e^{-j\frac{2\pi d}{\lambda }sin\theta },\ldots ,e^{-j\left(M-1\right)\frac{2\pi d}{\lambda }sin\theta }\right]}^{T}\\ & ={\left[1,e^{-j\pi sin\theta },\ldots ,e^{-j\left(M-1\right)\pi sin\theta }\right]}^{T}, \end{array}$$

where ${\left(\cdot \right)}^{T}$ denotes the transpose, and λ and d, respectively, denote signal wavelength and distance between two adjacent sensors.

The output of the beamformer is written as:

(3) $$y\left(k\right)=w^{H}x\left(k\right),$$

where $w={\left(w_{1},\ldots ,w_{M}\right)}^{T}$ is the complex beamformer weight vector, and ${\left(\cdot \right)}^{H}$ is the Hermitian transpose. The optimal beamformer weight vector, w, can be calculated by maximizing the output SINR, which is defined as follow:

(4) $$SINR≜\frac{\sigma_{0}^{2}{\left|w^{H}a_{0}\right|}^{2}}{w^{H}R_{i+n}w},$$

where $\sigma_{0}^{2}=E\left[{\left|s_{0}\left(k\right)\right|}^{2}\right]$ and $a_{0}$ denote the SOI power and SV, E[⋅] denotes the expectation operator of the stochastic variable. $R_{i+n}\in {\mathbb{C}}^{M\times M}$ denotes the precise INCM which can be written as:

(5) $$\begin{array}{ll} R_{i+n} & =E\left\{\left[x_{i}\left(k\right)+x_{n}\left(k\right)\right]{\left[x_{i}\left(k\right)+x_{n}\left(k\right)\right]}^{H}\right\}\\ & =\overset{L}{\underset{l=1}{\sum }}\sigma_{l}^{2}a_{l}a_{l}^{H}+E\left[x_{n}\left(k\right)x_{n}^{H}\left(k\right)\right]\\ & =R_{i}+\sigma_{n}^{2}I, \end{array}$$

where $\sigma_{l}^{2}=E\left[{\left|s_{l}\left(k\right)\right|}^{2}\right]$ and I denote the lth interference power and identity matrix, respectively, and $\sigma_{n}^{2}$ is the noise power. The main purpose of the optimal beamformer is to maximize the output SINR while keeping the SOI undistorted at the same time, which is the so-called the minimum variance distortionless response (MVDR) problem [2]:

(6) $$\underset{w}{\min}w^{H}R_{i+n}w\;s.t.w^{H}a_{0}=1.$$

The optimal beamformer weight vector is given by:

(7) $$w_{\mathrm{opt}}=\frac{R_{i+n}^{-1}a_{0}}{a_{0}^{H}R_{i+n}^{-1}a_{0}}.$$

In practical applications, the precise INCM $R_{i+n}$ is always unavailable, and it is usually replaced by SCM $\hat{R}$:

(8) $$\hat{R}=\frac{1}{K}\overset{K}{\underset{k=1}{\sum }}x\left(k\right)x^{H}\left(k\right),$$

where *K* denotes the number of snapshots. As K increases, $\hat{R}$ converge to the actual one. It has been proved that replacing $R_{i+n}$ by SCM $\hat{R}$ does not change the optimal output SINR [2]. Substituting the actual SOI SV $a_{0}$ by the nominal SV ${\bar{a}}_{0}$ based on the known array structure, the optimal weight vector becomes the sample covariance inversion (SMI) beamformer:

(9) $$w_{SMI}=\frac{{\hat{R}}^{-1}{\bar{a}}_{0}}{{\bar{a}}_{0}^{H}{\hat{R}}^{-1}{\bar{a}}_{0}}.$$

With the optimal weight vector, the Capon spatial power spectrum, is employed as a power estimator over all directions [28]:

(10) $$\begin{array}{cc} \hat{P}\left(\theta \right) & =w_{SMI}^{H}\hat{R}w_{SMI}\\ & =\frac{1}{{\bar{a}}^{H}\left(\theta \right){\hat{R}}^{-1}\bar{a}\left(\theta \right)}, \end{array}$$

where $\bar{a}\left(\theta \right)$ is the nominal SV associated with $\theta \in \left(-90^{\circ },90^{\circ }\right)$.

## 3. Proposed Method

The main idea of the proposed method is to utilize the reconstructed INCM and the corrected SOI SV to derive the optimal weight vector. Depending on the minimum mean square error criterion, a blocking matrix is obtained. The orthogonality of the subspace is employed to derive the projection matrix, and the INCM is reconstructed by replacing the eigenvector columns of the SCM such that they can span to the signal subspace with the corresponding eigenvectors of the projection matrix. The optimal weight vector is obtained along with the SOI SV estimated by the iterative mismatch approximation method.

### 3.1. INCM Reconstruction

Different from Capon power spectrum integration and interference estimation based INCM reconstruction methods, we present a blocking matrix based on the matrix filter principle in [36,37], which is suitable for suppressing signals illuminating within a specific angular region. Consider a blocking matrix $G\in {\mathbb{C}}^{M\times M}$, the property of G can be described as:

(11) $$G^{H}\bar{a}\left(\theta \right)=\{\begin{array}{r} \bar{a}\left(\theta \right),\;\;\theta \in \Theta_{p}\\ 0,\;\;\theta \in \Theta_{s} \end{array},$$

where $\Theta_{s}$ denotes the stopband angular region, and the direction of arrival (DOA) of the SOI lies in it. $\Theta_{p}$ denotes the passband angular region which contains the locations of interference. Sampling $\Theta_{s}$ and $\Theta_{p}$ uniformly with the $N_{s}$ and $N_{p}$ sampling points, the corresponding angular sequences can be presented as $\theta_{p}\;\left(p=1,\ldots ,N_{p}\right)$ and $\theta_{s}\;\left(s=1,\ldots ,N_{s}\right)$. The blocking matrix design problem based on the minimum mean square error criterion can be described as:

(12) $$\underset{G}{\min\|}\;G^{H}P-{\overset{˘}{P}\|}_{F},$$

where ${\left\|\cdot \right\|}_{F}$ denotes Frobenius norm. $P=\left[\bar{a}\left(\Theta_{p}\right),\bar{a}\left(\Theta_{s}\right)\right]\in {\mathbb{C}}^{M\times \left(N_{p}+N_{s}\right)}$ denotes the nominal manifold matrix, and $\overset{˘}{P}=\left[\bar{a}\left(\Theta_{p}\right),0_{M\times N_{s}}\right]\in {\mathbb{C}}^{M\times \left(N_{p}+N_{s}\right)}$ denotes the desired manifold matrix. The solution to (12) can be found by taking the gradient of $F\left(G\right)=G^{H}P-{\overset{˘}{P}}_{F}$ and making it equal to zero:

(13) $$\begin{array}{cc} \frac{\partial F\left(G\right)}{\partial G} & =\frac{\partial }{\partial G}tr{\left[{\left(G^{H}P-\overset{˘}{P}\right)}^{H}\left(G^{H}P-\overset{˘}{P}\right)\right]}^{\frac{1}{2}}=0\\ G & ={\left(PP^{H}\right)}^{-1}P{\overset{˘}{P}}^{H}. \end{array}$$

**Figure 1:**
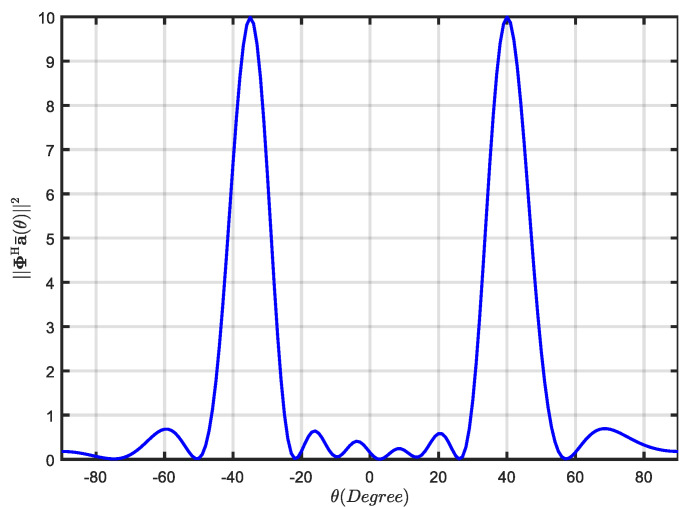
The value of $\|\Phi^{H}\bar{a}{\left(\theta )\right\|}^{2}$ versus θ.

Performing eigen decomposition on $\hat{R}$ yields:

(14) $$\begin{array}{ll} \hat{R} & =\sum_{i=1}^{M}\gamma_{i}u_{i}u_{i}^{H}=U\Gamma U^{H}\\ & =U_{s}\Gamma_{s}U_{s}^{H}+U_{n}\Gamma_{n}U_{n}^{H}, \end{array}$$

where $\gamma_{1}\geq \gamma_{2}\geq \cdots \gamma_{M-1}\geq \gamma_{M}$ denotes the eigenvalues of $\hat{R}$ arranged in descending order. The minimum eigenvalue can be approximately considered as the estimation of the noise power ${\tilde{\sigma }}_{n}^{2}$ [6]. $u_{i}\in {\mathbb{C}}^{M\times 1}$ is an eigenvector associated with $\gamma_{i}$. $U_{s}=\left(u_{1},\ldots ,u_{L+1}\right)\in {\mathbb{C}}^{M\times \left(L+1\right)}$ and $U_{n}=\left(u_{L+2},\ldots ,u_{M}\right)\in {\mathbb{C}}^{M\times \left(M-L-1\right)}$, respectively, denote the signal subspace eigenvectors and the noise subspace eigenvectors. $\Gamma_{s}=diag\left(\gamma_{1},\ldots ,\gamma_{L+1}\right)\in {\mathbb{C}}^{M\times \left(L+1\right)}$ and $\Gamma_{n}=diag\left(\gamma_{L+2},\ldots ,\gamma_{M}\right)\in {\mathbb{C}}^{M\times \left(M-L-1\right)}$ are diagonal matrices. According to the properties of the eigen subspace, we have:

(15) $$span\left\{u_{1},\ldots ,u_{L+1}\right\}=span\left\{a_{0},\ldots ,a_{L}\right\},$$

where $span\left\{u_{1},\ldots ,u_{L+1}\right\}$ denotes the spanned subspace generated by the vector group $\left\{u_{1},\ldots ,u_{L+1}\right\}$. Then, any accurate SV $a_{l}$ can be expressed as a linear combination of columns of $U_{s}$ [27], which means that the projection matrix $\Phi =U_{s}U_{s}^{H}G$ is orthogonal to $a_{0}$. $Q=\|\Phi^{H}\bar{a}{\left(\theta \right)\|}^{2}$ is used to measure the orthogonality between projection matrix Φ and $\bar{a}\left(\theta \right)$. Assume that the SOI and interference impinge on the half-wavelength spacing ULA with M=10 from $\theta_{0}=3^{\circ }$, $\theta_{1}=-35^{\circ }$, and$\;\theta_{2}=42^{\circ }$, the stopband and passband region are set as $\Theta_{s}=\left(\theta_{0}-6^{\circ },\theta_{0}+6^{\circ }\right)$ and $\Theta_{p}=\left(-90^{\circ },\theta_{0}-6^{\circ }\right){⋃}^{\;}\left(\theta_{0}+6^{\circ },90^{\circ }\right)$. It can be observed from Figure 1 that Q will be much smaller when $\theta =\theta_{0}$ than $\theta =\theta_{1,2}$. This means Φ and $\bar{a}\left(\theta_{0}\right)$ are orthogonal or approximately orthogonal. In addition, the value of Q corresponding to $\theta_{1,2}$ is equal to $\|\bar{a}{\left(\theta_{1,2}\right)\|}^{2}$. Hence, projection matrix Φ collects the spatial information of interference and removes the spatial information of the SOI.

Consider that the interference SV lies in the signal subspace spanned by the dominant eigenvectors of Φ. Then, employing eigen decomposition on Φ to obtain the signal subspace:

(16) $$\Phi =B\Lambda B^{H}=B_{s}\Lambda_{s}B_{s}^{H}+B_{n}\Lambda_{n}B_{n}^{H},$$

where $B=\left[b_{1},\ldots ,b_{M}\right]=\left[B_{s},B_{n}\right]$ and $\Lambda =diag\{\lambda_{1},\ldots ,\lambda_{M})$, respectively, denote unitary and diagonal matrices. $b_{l},l=1,\ldots ,M$ denotes the eigenvector corresponding to $\lambda_{l}$, which are arranged in descending order. In addition, $B_{s}$ contains L+1 eigenvectors columns corresponding to the L+1 largest eigenvalues, and $span\left\{b_{1},\ldots ,b_{L+1}\right\}$ can be considered as the signal subspace.

Substituting $B_{s}$ back into (14) and replacing $U_{s}$, then we can obtain the reconstructed INCM:

(17) $${\tilde{R}}_{i+n}=B_{s}\Gamma_{s}B_{s}^{H}+U_{n}\Gamma_{n}U_{n}^{H},$$

plotting the power spectrum of ${\tilde{R}}_{i+n}$ by replacing ${\tilde{R}}_{i+n}$ with $\hat{R}$ in (10), which is written as:

(18) $$\tilde{P}\left(\theta \right)=\frac{1}{{\bar{a}}^{H}\left(\theta \right){\tilde{R}}_{n+i}^{-1}\bar{a}\left(\theta \right)}.$$

**Figure 2:**
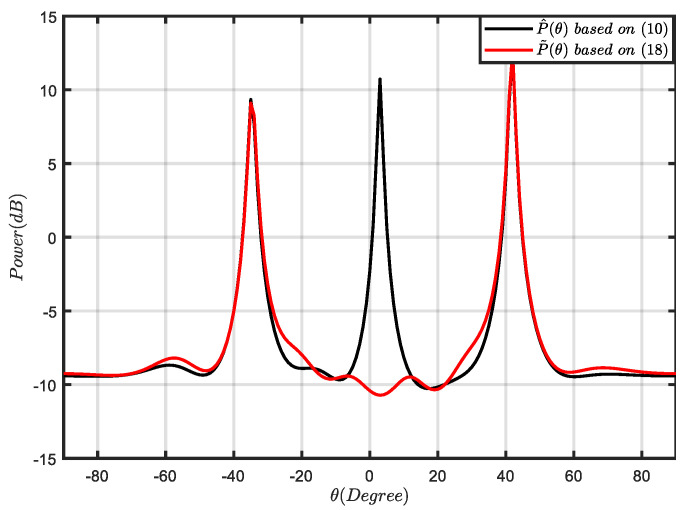
Comparison of the Capon power spectrum (10) and the power spectrum based on (18) with SNR=30 dB, INR=20 dB, and K=100.

The spatial power spectrum distribution based on (10) and (18) is drawn in Figure 2. The SOI was assumed to be impinging from $\theta_{0}=3^{\circ }$ with a fixed SNR=30 dB and that two interferences were impinging from $\theta_{1}=-35^{\circ }$ and $\theta_{2}=42^{\circ }$ with a fixed interference-to-noise rate (INR) INR=20 dB, respectively. This shows that the blocking matrix can effectively filter the SOI components and interference components are retained. Therefore, ${\tilde{R}}_{i+n}$ can be used as an INCM to derive the beamformer.

### 3.2. SOI SV Estimation and Beamformer Weight Vector Calculation

For estimating the SOI SV, we employed the iterative mismatch approximation method proposed in [29] to correct the presumed SOI SV. The iterative mismatch approximation method depends on searching for the SV mismatch in the margin of the amplitude and phase error. In the presence of SV mismatches, the actual SOI SV can be written as $a_{0}={\bar{a}}_{0}+e=\alpha \circ {\bar{a}}_{0}\circ e^{j\beta }$. Employing the principle of estimating the signal steering vectors via maximizing the beamformer output power [16], the estimated SOI SV ${\tilde{a}}_{0}$ can be obtained by solving the pseudo-optimization problem:

(19) $$\begin{array}{cc} \underset{\alpha ,\beta }{\max}\;\tilde{P}\left(\theta_{q},\alpha ,\beta \right)\\ s.t.\;\;\;\;\left|1-\alpha_{m}\right| & \leq \epsilon \\ \left|\beta_{m}\right| & \leq \phi , \end{array}$$

where ∘ denotes the Hadamard product, and the power spectrum associated with $\theta_{q},\alpha ,\beta$ is written as $\tilde{P}\left(\theta_{q},\alpha ,\beta \right)=1/\left(\alpha \circ \bar{a}(\theta_{q}\right)\circ e^{j\beta }{)}^{H}{\hat{R}}^{-1}\left(\alpha \circ \bar{a}\left(\theta_{q}\right)\circ e^{j\beta }\right)$. ϵ and ϕ denote the predefined boundary values of the amplitude and phase mismatch, respectively. $\alpha ={\left(\alpha_{1},\ldots ,\alpha_{M}\right)}^{T}$ and $\beta ={\left(\beta_{1}\ldots ,\beta_{M}\right)}^{T}$ denote the amplitude and phase mismatch vectors, respectively. The iterative mismatch approximation method is described in Algorithm 1. In addition, the initial nominal SOI SV is associated with the middle value of $\Theta_{s}$.


**Algorithm 1.** Iterative mismatch approximation method.
**Input: **$\epsilon ,\;\phi ,{\bar{a}}_{q},{\hat{R}}^{-1}$ **Output:** ${\tilde{a}}_{q},pwr$ 1:  Initialize $a={\bar{a}}_{q}$ 2:  **for** it1=1…M 3:   ${\tilde{\Theta }}_{\alpha }=\left[\alpha_{ql},\alpha_{qh}\right]=\left[1-\epsilon ,\;1+\epsilon \right]$**,**${\tilde{\Theta }}_{\beta }=\left[\beta_{ql},\beta_{qh}\right]=\left[e^{j\left(-\phi \right)},e^{j\left(\phi \right)}\right]$ 4:   **for**it2=1…depth 5:    $E_{ap}={\tilde{\Theta }}_{\beta }⊗{\tilde{\Theta }}_{\alpha },\;E_{ap}\in {\mathbb{C}}^{1\times 4}$ 6:    $a_{i}\left(it1\right)={\bar{a}}_{q}\left(it1\right)E_{gp}\left(i\right),i=1,\ldots ,4$ 7:    Calculate p(i) by substituting $a_{i}$ into Equation (10) 8:    (pwr,idx)=max(p) 9:    Zoom out of the amplitude/phase error area built by ${\tilde{\Theta }}_{\alpha }$ and ${\tilde{\Theta }}_{\beta }$ to $E_{ap}\left(idx\right)$ 10:    Update ${\tilde{\Theta }}_{\alpha }$ and ${\tilde{\Theta }}_{\beta }$ 11:   **end** 12:    ${\tilde{a}}_{q}\left(it1\right)={\bar{a}}_{q}\left(it1\right)E_{ap}\left(idx\right)$ 13:  **end**

Then, the estimated SOI SV ${\tilde{a}}_{0}$ can be corrected as ${\tilde{a}}_{q}$. Substituting (17) together with ${\tilde{a}}_{0}$ back into the Capon beamformer (7), the robust adaptive beamforming based on INCM reconstruction via the projecting matrix and SV estimation can be written as:

(20) $$\tilde{w}=\frac{{\tilde{R}}_{i+n}^{-1}{\tilde{a}}_{0}}{{\tilde{a}}_{0}^{H}{\tilde{R}}_{i+n}^{-1}{\tilde{a}}_{0}}.$$

In a general case, the estimated SOI SV may be imprecise. Hence, we took ${\tilde{a}}_{0}$ as an input parameter and performed multiple iterations to improve accuracy. The iteration was terminated when the following conditions were satisfied:

(21) $$\left|\frac{{\tilde{\sigma }}_{q}^{2}{|}_{current}-{\tilde{\sigma }}_{q}^{2}{|}_{previous}}{{\tilde{\sigma }}_{q}^{2}{|}_{previous}}\right|<\varphi ,$$

where φ and ${\tilde{\sigma }}_{q}^{2}$ denote a predefined saturation coefficient and power corresponding to ${\tilde{a}}_{q}$, respectively. The method we propose is summarized in Algorithm 2.
**Algorithm 2.** Proposed RAB method.**1:** Calculate the SCM $\hat{R}$ using (8) and eigen decompose $\hat{R}$ to obtain $U_{S}$ and ${\tilde{\sigma }}_{n}^{2}$; **2:** Obtain the blocking matrix G using (13) and the projection matrix $\Phi =U_{s}U_{s}^{H}G$**;** **3:** Eigen decompose Φ to obtain $B_{s}$ and reconstruct INCM via (17); **4:** Using the iterative mismatch approximation method in Algorithm 1 to estimate the SOI SV; **5**: Substitute
${\tilde{R}}_{i+n}$ and ${\tilde{a}}_{0}$ back into (20) to obtain the weight vector.


The number of floating-point operations (flops) was employed to measure the computational complexity. The computational complexity of the proposed method is mainly determined by calculating the SCM $\hat{R}$ and matrix eigen decomposition, calculating G and estimating SOI SV ${\tilde{a}}_{0}$. Calculating the SCM $\hat{R}$ costs approximately $O\left(M^{2}K\right)$ flops. Calculating G costs approximately $O\left(N_{S}M^{2}+N_{P}M^{2}+2M^{3}\right)$ flops. The computational complexity of matrix inversion and eigen decomposition is approximately $O\left(M^{3}\right)$ flops. Assuming that depth=D, the computational complexity of estimating the SOI SV would be approximately$\;O\left(M^{3}+3DM\right)$ flops. In practice, calculating G is independent of $\hat{R}$ and can be seen as a pre-processing operation of the proposed method. With K≫M≅D, the overall complexity of proposed method is approximately $O\left(M^{2}K\right)$ flops. In [6], the major computations were conducted to solve a QCQP problem and estimate interference SVs. Suppose that S denotes the number of search points in $\bar{\Theta }$, the computational complexity is $O\left(max\left(M^{2}S,M^{3.5}\right)\right)$. In [30], the sample points, N, for the line search significantly affected the adjustment of the SVs. Hence, when N>M, the computational complexity is $O\left(LNM^{2}\right)$. The computational complexity of the MEPS-IPNC in [31] was $O\left(M^{2}L\right)$, where L is a small multiple of M. In [32], the computational complexity was $O\left(max\left(M^{2}J,M^{2}S\right)\right)$, where S and J are the number of the sampling points in $\Theta_{s}$ and $\bar{\Theta_{s}\cup^{\;}\Theta_{i}}$. In [33], the computational complexity was declared to be $O\left(M^{3}\right)$. In [34], the computational complexity was $O\left(max\left(M^{2}J,M^{2}S\right)\right)$, where S and J are the number of the sampling points in Θ and $\bar{\Theta }$. In [35], the computational complexity was declared to be $O\left(M^{2}I\left(L+1\right)\right)$, where I is the number of sample points for Θ and $\Theta_{i-l}$. Clearly, since the power spectrum calculation, power spectrum integration, and QCQP problem solving are avoided in our proposed method, it has the lowest computational complexity. Furthermore, prior information regarding the SOI angular region and array geometry are needed.

## 4. Simulation

In this section, a half-wavelength spacing ULA with M=10 was considered. We assumed that there existed an SOI impinging from the direction of $\theta_{0}=3^{\circ }$ and two interferences impinging from $\theta_{1}=-35^{\circ }$ and $\theta_{2}=42^{\circ }$. The additive noise was presumed to be a complex circularly symmetric Gaussian zero-mean unit-variance spatially and temporally white process. All these sources were narrowband and assumed to be independent to the noise. To obtain each output SINR point, 200 Monte Carlo trials were used in each simulation. The proposed method was compared with the RAB method based on INCM reconstruction and steering vector estimation (INCM-SVE) in [6], subspace-decomposition and SV adjustment (SDA) in [30], MEPS-IPNC in [31], INCM reconstruction via orthogonality of subspace (INCM-OS) in [32], desired signal eigenvalue replacement (DSEB) in [33], SPCMR in [34] and INCM reconstruction via the intersection of subspaces (INCM-IS) in [35]. For all methods involved in the comparisons, the angular region was presumed to be $\Theta =\Theta_{s}=\left(\theta_{0}-6^{\circ },\theta_{0}+6^{\circ }\right)\;\mathrm{and}\;\bar{\Theta }=\Theta_{p}=\left(-90^{\circ },\theta_{0}-6^{\circ }\right){⋃}^{\;}\left(\theta_{0}+6^{\circ },90^{\circ }\right)$. and the interference angular region to be $\Theta_{i}=\left(\theta_{i}-6^{\circ },\theta_{i}+6^{\circ }\right)$. The number of non-dominant eigenvectors of the matrix C was set as L=7, and the RCB boundary was $\epsilon =\sqrt{0.1}$ in [6]. The N=7 dominant eigenvectors of matrix B were employed for $B_{1}$ in [32]. ξ=0.95 as in [33]. The constant satisfying μ=0.9 and $\tau =\sqrt{0.1}$ were as in [34]. Sampling points L=5M and S=20 were as in [31]. The scale factor $\tilde{\mu }=0.1$ and the sampling points $N=2\tilde{\mu }/0.01$ were as in [30]. For the proposed method, the amplitude and phase mismatch boundary were set as ϵ=0.3 and $\phi =6^{\circ }$, respectively, the depth of the iteration was set as depth=10, and the saturation value was φ=0.05. Furthermore, the MATLAB CVX toolbox was used to solve the QCQP optimization problem in [6]. In our simulations, the optimal output SINR can be calculated by:

(22) $$SINR_{opt}=\sigma_{0}^{2}a_{0}^{H}R_{i+n}^{-1}a_{0}.$$

### 4.1. Example 1: Mismatch Due to the Amplitude and Phase Error of the SV

**Figure 3:**
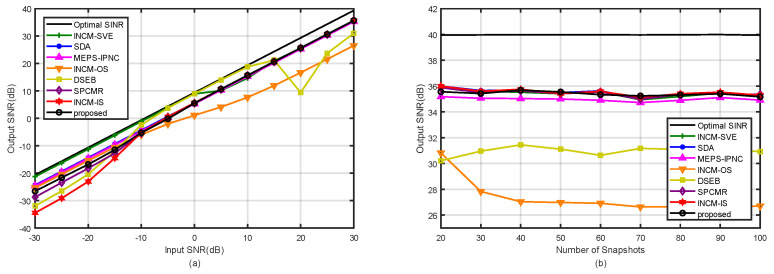
Output SINRs in the case of amplitude and phase errors versus (**a**) input SNR with K=100; (**b**) the number of snapshots with SNR=30 dB.

In the first example, the influence of the SVs with arbitrary amplitude and phase errors on the beamformer output SINR was considered. The relationship between the mth element of the nominal SV and the actual SV was modeled as $a_{m}=\alpha_{m}{\bar{a}}_{m}e^{j\beta_{m}}$, where the arbitrary amplitude error, $\alpha_{m}$, and phase error, $\beta_{m}$, on each array sensor, respectively, followed the Gaussian distribution $N\left(1,0.05^{2}\right)$ and $N\left(0,{\left(5^{\circ }\right)}^{2}\right)$ [6]. Figure 3a depicts the output SINR of the tested methods versus the input SNR for the fixed number of snapshots K=100. It was observed that the proposed method had a similar performance among the tested methods except in [32,33] at high SNRs. In addition, the performance of the proposed method was only lower than in [6] when the SNR was low. However, the computational complexity of our method was obviously lower than that in [6]. In Figure 3b, the output SINRs are shown versus the number of snapshots for the fixed SNR=30 dB and INR=20 dB. The proposed method had a similar performance to the tested methods in [6,30,34,35], and the number of snapshots did not affect the performance of our proposed method.

### 4.2. Example 2: Mismatch Due to the Random Look Direction Error

**Figure 4:**
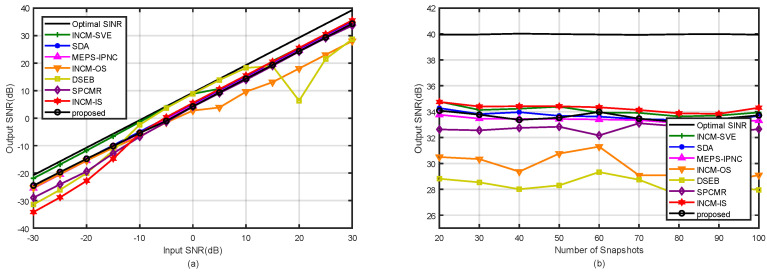
Output SINRs in the case of the look direction error versus (**a**) input SNRs with K=100; (**b**) the number of snapshots with SNR=30 dB.

In the second example, the influence of the random look direction errors on the beamformer output SINR was considered. Assuming that the look direction mismatch of both the SOI and interferences were uniformly distributed in ($-5^{\circ },5^{\circ })$. That is means that the DOA of the SOI was uniformly distributed in ($-2^{\circ },8^{\circ })$, and the DOAs of the two interference were uniformly distributed in ($-40^{\circ },30^{\circ })$ and ($37^{\circ },47^{\circ })$. Note that the random DOAs of the SOI and interferences changed in each trial while remaining constant over snapshots. Figure 4a shows the output SINRs of the tested methods versus the input SNRs with the fixed snapshots K=100. It was observed that our proposed method was only inferior to that in [6] in the performance at low SNR and inferior to that in [6,35] at high SNRs. Figure 4b depicts the output SINRs of the tested methods against the snapshot number at SNR=30 dB and INR=20 dB. It was observed that the performance of our proposed method was similar to that in [30] when K>40. In addition, the methods in [33,34] were significantly affected by mismatches due to the look direction error.

### 4.3. Example 3: Mismatch Due to the Incoherent Local Scattering Error

In the third example, the influence of the incoherent local scattering error on the beamformer output SINRs was considered. The SOI was assumed to have a time-varying signature, which was modeled as:

(23) $${\hat{x}}_{s}\left(k\right)=s_{0}\left(k\right)a_{0}+\overset{4}{\underset{p=1}{\sum }}s_{p}\left(k\right)\bar{a}\left(\theta_{p}\right),$$

where $a_{0}$ denotes the SOI SV. $\bar{a}\left(\theta_{p}\right)\left(p=1,2,3,4\right)$ denotes the incoherent scattering signal SV, and the DOAs, $\theta_{p}$, are independently distributed in a Gaussian distribution drawn from a random generator $N\left(\theta_{0},4^{\circ }\right)$ in each trial. $s_{p}\left(k\right)$ are independently and identically distributed zero-mean complex Gaussian random variables independently drawn from a random generator, N(0,1). In addition, $\theta_{p}$ changes from trial to trial, while it remains fixed over the samples. At the same time, $s_{p}\left(k\right)$ changes both from trial to trial and from sample to sample. In this case, the SOI covariance matrix is no longer a rank-one matrix and the output SINR should be expressed as [5]:

(24) $$SINR_{opt}=\frac{w^{H}R_{s}w}{w^{H}R_{i+n}w}.$$

**Figure 5:**
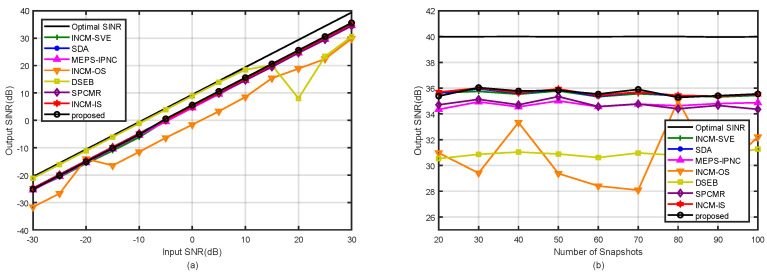
Output SINRs in the case of incoherent local scattering error versus (**a**) input SNRs with K=100; (**b**) the number of snapshots with SNR=30 dB.

The optimal weight vector can be obtained by maximizing the SINR [5]:

(25) $$w_{opt}=P\left\{R_{i+n}^{-1}R_{s}\right\},$$

where *P*{·} denotes the principal eigenvector of a matrix. Figure 5a shows the output SINRs of the tested methods versus the input SNRs with the fixed snapshots K=100. It was observed that the performance of our proposed method was similar to that in [6,30] at high SNRs and only lower than in [33] at low SNRs. However, the method in [33] had severe performance degradation at high SNRs. Figure 5b depicts the output SINRs of the tested methods against the snapshot number at SNR=30 dB and INR=20 dB. It was observed that the proposed method had a similar performance with the tested methods in [6,30,35], and the number of snapshots did not affect the performance of our proposed method.

### 4.4. Example 4: Mismatch Due to the Coherent Local Scattering Error

**Figure 6:**
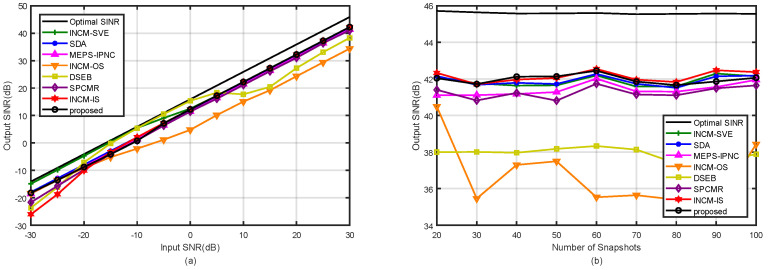
Output SINRs in the case of coherent local scattering error versus (**a**) input SNR with K=100; (**b**) the number of snapshots with SNR=30 dB.

In the fourth example, the influence of the coherent local scattering mismatch on the beamformer output SINRs was considered. The coherent local scattering mismatch usually occurs in multipath propagation scenarios. Assume that the SOI is distorted by local scattering and consists of four coherent paths; the actual SV is formed as:

(26) $${\hat{a}}_{0}=a_{0}+\overset{4}{\underset{p=1}{\sum }}e^{j\phi_{p}}\bar{a}\left(\theta_{p}\right),$$

where $a_{0}$ denotes the SOI SV. $\bar{a}\left(\theta_{p}\right)\left(p=1,2,3,4\right)$ denotes the coherent signal path from $\theta_{p}$. $\theta_{p}$ are independently distributed in a Gaussian distribution drawn from a random generator, $N\left(\theta_{0},4^{\circ }\right)$, in each trial. $\phi_{p}$ denotes the path phase and uniformly distributed in (0,2π) from trial to trial. $\theta_{p}$ and $\phi_{p}$ change from trial to trial, while it remains fixed over the samples. Figure 6a shows the output SINRs of the tested methods versus the input SNRs with the fixed snapshots K=100. It was observed that the performance of the optimal beamformer had an approximately 6 dB increment in output SINRs due to the extra paths. The performance of our proposed method was similar to that in [6,30,35] at high SNRs. The method in [6] achieved the best performance at the cost of the highest complexity compared with the others. Figure 6b depicts the output SINRs of the tested methods against the snapshot number at SNR=30 dB and INR=20 dB. It was observed that the proposed method had a small impact on the number of snapshots.

## 5. Conclusions

In this paper, we proposed a low-complexity RAB method based on INCM reconstruction via subspace projection. In this method, the component of the SOI in the SCM was eliminated by replacing the eigenvector columns in the SCM such that they could span to the signal subspace with the corresponding eigenvectors in the projection matrix. Meanwhile, the SOI SV was estimated by employing the iterative mismatch approximation method. Since the calculation of the blocking matrix can be seen as pre-processing, the complexity of INCM reconstruction only depends on a countable number of matrices’ eigen decomposition and multiplication. Both the analysis and simulation illustrate that the proposed method is robust to various types of mismatches while maintaining low complexity.

## Data Availability

Not applicable.
